# Evaluation of delivery of enteral nutrition in mechanically ventilated Malaysian ICU patients

**DOI:** 10.1186/1471-2253-14-127

**Published:** 2014-12-23

**Authors:** Keng F Yip, Vineya Rai, Kang K Wong

**Affiliations:** Department of Anaesthesiology, Faculty of Medicine, University of Malaya, 50603 Kuala Lumpur, Malaysia

**Keywords:** Enteral nutrition, Gastric residual volume, Interruptions to feeding

## Abstract

**Background:**

There are numerous challenges in providing nutrition to the mechanically ventilated critically ill ICU patient. Understanding the level of nutritional support and the barriers to enteral feeding interruption in mechanically ventilated patients are important to maximise the nutritional benefits to the critically ill patients. Thus, this study aims to evaluate enteral nutrition delivery and identify the reasons for interruptions in mechanically ventilated Malaysian patients receiving enteral feeding.

**Methods:**

A cross sectional prospective study of 77 consecutive patients who required mechanical ventilation and were receiving enteral nutrition was done in an open 14-bed intensive care unit of a tertiary hospital. Data were collected prospectively over a 3 month period. Descriptive statistical analysis were made with respect to demographical data, time taken to initiate feeds, type of feeds, quantification of feeds attainment, and reasons for feed interruptions. There are no set feeding protocols in the ICU. The usual initial rate of enteral nutrition observed in ICU was 20 ml/hour, assessed every 6 hours and the decision was made thereafter to increase feeds. The target calorie for each patient was determined by the clinician alongside the dietitian. The use of prokinetic agents was also prescribed at the discretion of the attending clinician and is commonly IV metoclopramide 10 mg three times a day.

**Results:**

About 66% of patients achieved 80% of caloric requirements within 3 days of which 46.8% achieved full feeds in less than 12 hours. The time to initiate feeds for patients admitted into the ICU ranged from 0 – 110 hours with a median time to start feeds of 15 hours and the interquartile range (IQR) of 6–59 hours. The mean time to achieve at least 80% of nutritional target was 1.8 days ± 1.5 days. About 79% of patients experienced multiple feeding interruptions. The most prevalent reason for interruption was for procedures (45.1%) followed by high gastric residual volume (38.0%), diarrhoea (8.4%), difficulty in nasogastric tube placement (5.6%) and vomiting (2.9%).

**Conclusion:**

Nutritional inadequacy in mechanically ventilated Malaysian patients receiving enteral nutrition was not as common as expected. However, there is still room for improvement with regards to decreasing the number of patients who did not achieve their caloric requirement throughout their stay in the ICU.

## Background

Nutrition in the Intensive Care Unit (ICU), though seemingly elementary, presents with varied number of challenges. Patients admitted in the ICU are often multidisciplinary and heterogeneous in terms of general condition, severity of illness and specific needs. Differences in opinions amongst care providers add to the vast variation in debatable topics, especially in nutritional therapy. In critically ill patients, enteral nutrition is preferred in the majority of cases because of its ease of use, reduced cost and lower risk of catheter-related septic complications [1]. However, inadequate nutritional intake in enterally fed patients remains a global issue [2, 3]. A recent study by Miroslav [4], revealed that 26% of feeding interruptions in a Boston teaching hospital were avoidable and as such led to a 30% increase in length of stay in the ICU and a 50% increase length of hospital stay in patients. Several factors that contribute to inadequate nutritional delivery include, but not restricted to, gastrointestinal intolerance, displacement or obstruction of the feeding tube, therapeutic procedures, airway management or nursing procedures [5].

The current ICU in our tertiary care institution does not have an Enteral Nutrition Protocol and feeding has been administered as ordered by the care provider. Current findings have already shown that the presence of an enteral feeding protocol is associated with significant improvements in nutritional practice [6, 7]. As our current ICU does not have a standard feeding protocol, we felt that nutritional goals such as the time to initate and time to achieve full feeds would be slow to be met and there will be a high number of feeding interruptions. To date, no studies have reported on the nutritional status of critically ill adult Malaysian patients on mechanical ventilation receiving enteral nutrition. Thus, this study aims to evaluate enteral nutrition delivery and identify the reasons for interruptions in mechanically ventilated Malaysian patients receiving enteral feeding.

## Methods

A cross sectional prospective study was conducted in an open 14-bed general intensive care unit at a tertiary care hospital for a period of three months. Patients were included in the study if they were above 18 years of age, require mechanical ventilation, expected to stay for at least 24 hours in the ICU and received enteral nutrition at any time while being ventilated. Patients were excluded if enteral nutrition was clinically contraindicated. Contraindications to enteral feeding includes, exclusively: (1) severe hemodynamic instability; (2) bowel obstruction; (3) severe protracted ileus; (4) major upper gastrointestinal bleeding; (5) intractable vomiting or diarrhoea; and (6) gastrointestinal ischaemia. The patients were individually observed until discontinuation of enteral feeding, discharged from the ICU or death. This study was approved by Medical Ethics Committee of University Malaya Medical Centre, Lembah Pantai 59100 Kuala Lumpur. Consent was waived as this is an observational study.

Our primary endpoint was the time to achieve full feeds and secondary endpoints were the time to initiate feeds and reasons for interruption to feeding.

A multidisciplinary team which included an intensivist, registrars, registered nurses and dieticians were responsible for decisions related to patient care, time of insertion of feeding tube, initiation of enteral feeds, amount of feeds to be delivered and interruptions. Demographic data, time of admission, time of initiation of prescribed feeds, type of enteral formula, daily calories delivered, reasons of interruptions, amount of gastric residual volume, development of aspiration or infection complication rates and simplified acute physiology score (SAPS) II were recorded.

Energy intake was calculated using a goal of 25 kcal/kg/day. Patient’s weight was determined using a scale or ideal body weight if actual body weight could not be determined. If a patient's current body weight is greater than 120% of his ideal body weight, an adjusted body weight was used for the calculation:

Adjusted body weight = (current body weight – ideal body weight) × 0.25 + ideal body weight.

SAPS II was calculated for each patient at the time of enrolment. On each subsequent study day, the investigator included recorded data of the previous 24 hours.

### Statistical analysis

Statistical analyses were performed using the Statistical Package for the Social Sciences, Version 17.0 (SPSS Inc., Chicago, IL, USA). A *p* value of < 0.05 was considered to be statistically significant. A descriptive analysis was performed for the data collected. Continuous variables were summarized using means and standard deviations.

## Results

A total of 77 patients who fulfilled the inclusion criteria were recruited in this study. The patients’ demographic characteristics are shown in Table 1. Mean weight of the sample population obtained was 68.2 kg, with the mean weight of female and male patients being 63.49 kg (BMI 26.2) and 70.87 kg (BMI 24.7) respectively. A mean SAPS score of 40 was documented in patients who remained in the ICU after 24 hours. Patients were fed with Glucerna®, Nepro®, Osmolite®, Peptamen® or Pulmocare®. The time taken to initiate enteral feeding was between 0 and 110 hours. The median time to initiate was 15 hours (IQR 6–59 hours). The mean time of patients who received full feeds was 1.8 days ± 1.5 days. Twelve out of the 77 patients (15.6%) did not achieve full feeds over their stay in the ICU while 36 patients achieved full feeds within 12 hours of ICU admission (Table 2). Further analysis showed that 66% patients achieved 80% of caloric requirements within three days in ICU. During this period, interruptions to feeding were documented for all recruited patients. An interruption of one hour or more was considered significant and the reason for interruption was noted. Only 16 out of the 77 patients (20.7%) did not have any interruptions to feeds while the rest had one or more feeding interruptions. Out of 72 interruptions encountered during the study, 32 of them were due to clinical procedures/interventions on the patients (Table 3). These included planned extubations, tracheostomies, surgical interventions or radiological imaging. The rest were due to perceived high GRV by the attending clinician. There were no cases of aspirations reported.

**Table 1:** **Patient’s characteristics**

Characteristics	Value, N = 77
Age, mean (SD), years	51.0 (16.6)
Sex, n (%)	
Male	49 (63.6)
Female	28 (36.4)
Ethnicity, n (%)	
Malay	35 (45.5)
Chinese	22 (28.6)
Indian	16 (20.8)
Others	4 (5.2)
Weight, mean (SD), kg	68.2 (11.8)
SAPS II, range (SD)	11-78 (16.4)
Time to initiation of Enteral Feeding after ICU admission, median (IQR) hours	15 (6–59)
Days to full feed, mean (range), days, n = 65	1.8 (0–6)

All values are calculated based on a total of 77 patients unless otherwise stated.

ICU = intensive care unit; SAPS II = simplified acute physiology score II; SD = standard deviation.

**Table 2:** **Time taken for patients to achieve full feeds**

Time	n (%)
Less than 12 hours	36 (46.8)
More than 12 hours	29 (37.7)
Did not achieve full feeds	12 (15.6)

**Table 3:** **Reasons for interruptions of enteral feeding**

Reason	n (%)	
Procedures	32	(45.1)
High gastric residual volume (GRV)	27	(38.0)
300 ml	4	(14.8)
200 – 300 ml	14	(51.9)
200 ml	9	(33.3)
Diarrhoea	6	(8.4)
Vomiting	2	(2.9)
Difficulty in nasogastric (NG) tube placement	5	(5.6)

## Discussion

To the best of our knowledge, this is the first study to evaluate the delivery of enteral nutrition and the reasons for interruption in critically ill Malaysian patients receiving mechanical ventilation. The patients admitted to the ICU in our study generally reflected the Malaysian multiracial demeanour.

Majority were Malay (45.5%), followed by Chinese (28.6%) and Indian (20.8%). The European Society for Clinical Nutrition and Metabolism (ESPEN) recommends caloric intake of 20–25 kcal/kg/day during the catabolic phase and up to 25-30 kcal/kg/day during the anabolic phase [1]. The American Society for Parenteral and Enteral Nutrition (ASPEN) similarly advocates the energy requirement for critically ill adult to be 25–30 kcal/kg/day [8]. Although one study showed an increase in requirement in the second week of illness [9], there is not enough evidence to validate its use in this study. Stapleton et al. showed a positive association between moving closer to caloric goals and better clinical outcome [10]. Failure to achieve > 25% of caloric goals may increase the risk of nosocomial bloodstream infections [11, 12]. About 47% of patients achieved the full caloric requirement within the first 12 hours of ICU admission.

This suggests that almost half of our patients achieved full feeds in less than 24 hours. At three days, 66% of the patients achieved their full caloric requirements. A study by Kim et al. [13] on the adequacy of early enteral nutrition in Korean adult patients found that about two-thirds of their patients failed to meet 90% of their energy requirements during the first four days after initiation of enteral nutrition and more than half of the patients received less than 90% of protein requirements during the study period. A study by O’Leary-Kelley et al. [3] also found that 68% of their mechanically ventilated patients were underfed. Compared to these studies, our results indicated a higher number of patients achieving their caloric requirements. Despite the absence of an Enteral Nutrition Protocol, the multidisciplinary team of this tertiary hospital which comprised of an intensivist, registrars, nurses and dieticians who are responsible for the mechanically ventilated patient’s needs may have contributed to 66% of the patients meeting their nutritional requirement goal in three days. Further studies are required to assess if a development of a feeding protocol would further increase the number of patients achieving their nutrition target in our population, as have been proven successful by Mackenzie et al. [7] Our data showed a large range in time (0 – 110 hours) to initiate feeds for patients admitted into the ICU, with a median time to start feeds of 15 hours (IQR 6–59 hours). A study by Rice et al. [14] reported that the average time to initiate enteral feeding after beginning mechanical ventilation was about two days. According to ESPEN 2006 guideline [1], feeds should be started within the first 24 hours of admission to the ICU. However, initiating enteral feeds within 24 hours of mechanical ventilation may be difficult as some patients may have other acute problems that first need to be treated

The time taken to achieve full feeds has its implication on the overall morbidity of patients in the sense that an escalating energy deficit has shown to be associated with increased mortality and morbidity [15, 16]. However, nutrition inadequacy cannot be attributed only to inadequate delivery but is confounded by the fact that critically ill patients are frequently in hyper-metabolic and catabolic states [17]. Simple modifiable factors such as type of feeds, early enteral feeds [18], reductions in feed interruptions [19] and a feeding protocol [6] have been postulated to improve delivery of nutrition and prevent complications related to underfeeding such as weakness, infections, increased length of ventilator days and mortality [20].

Procedures such as planned extubations, tracheotomies and radiological imaging were the most common reason that led to feed interruptions in these studies. Similar findings were reported by others [14, 21–24]. Little change or improvement can be done for this group of patients to reduce the duration and number of interruptions. Careful evaluation and implementation of fasting time should be made on a case by case basis and decisions should be made based on the type of procedure and surgical (abdominal, airway or peripheral) or imaging requirements at the discretion of the ICU consultant.

The next most common reason for feed interruption is high gastric residual volume (GRV). There was no significant difference in gastrointestinal intolerance in patients with GRV greater than 300 ml compared with those less than 300 ml. No difference in adverse outcomes was previously reported when GRV was increased to 500 ml [25]. The majority of feed cessation in this group was for GRV volume of 200–300 ml (51.9%). In recent years, there appears to be an acceptance of high GRV [19]. The recommendations were based on the assumption that most GRV would be aspirated to avoid production of less favourable outcome. As for this study, no case of aspiration was reported in all of the 77 patients. Our results are consistent with other studies that demonstrated gastrointestinal intolerance as a common reason for interruption in enteral feeding [5, 26, 27]. The high percentage of stoppages even for GRV <200 ml (33.3%) is observed to be attributed to the lack of feeding protocol in our ICU as well as the inexperience and lack of knowledge by some clinicians regarding newer studies on GRV. As this is a teaching hospital, most of the ad hoc clinical decisions are made by trainees.

The limitations of this study should be acknowledged. Our results reflect practices of a single unit in a single institution. Convenience sampling of subjects receiving enteral nutrition during a certain period may also not accurately represent characteristics of a larger sample. Therefore the results of this study should be interpreted with caution and should not be generalized to the wider population of patients on mechanical ventilation receiving enteral nutrition in Malaysia.

## Conclusion

In conclusion, although enteral feeds are started within a mean time of 22.5 hours and full feeds achieved early, there is still room for improvement to increase the number of patients who achieve their caloric requirement during their stay in the ICU.

## Abbreviations

ICU: Intensive care unit
GRV: Gastric residual volume
SAPS: Simplified acute physiology score
ESPEN: The European Society for Clinical Nutrition and Metabolism
VAP: Ventilator associated pneumonia.
